# Obesity and breast cancer prognosis: pre-diagnostic anthropometric measures in relation to patient, tumor, and treatment characteristics

**DOI:** 10.1186/s40170-023-00308-0

**Published:** 2023-06-27

**Authors:** Sixten Harborg, Maria Feldt, Deirdre Cronin-Fenton, Marie Klintman, Susanne O. Dalton, Ann H. Rosendahl, Signe Borgquist

**Affiliations:** 1 Department of Oncology, Aarhus University/Aarhus University Hospital, Entrance C, Level 1, C106, Palle Juul-Jensens Boulevard 99, 8200 Aarhus N, Denmark; 2 Department of Clinical Epidemiology, Aarhus University, Aarhus, Denmark; 3 Department of Clinical Sciences Lund, Oncology, Lund University, Skåne University Hospital, Lund, Sweden; 4 Survivorship and Inequality in Cancer, Danish Cancer Society Research Center, Copenhagen, Denmark; 5 Department of Clinical Oncology & Palliative Services, Zealand University Hospital, Næstved, Denmark

## Abstract

**Purpose:**

Examine the association between obesity and clinical outcomes in early breast cancer and assess if patient, tumor, and treatment characteristics modify such associations in Malmö Diet and Cancer Study patients (MDCS).

**Methods:**

The MDCS enrolled 17,035 Swedish women from 1991 to 1996. At enrollment, participants' body mass index (BMI), waist circumference and body fat percentage measures were collected. We identified all female MDCS participants with invasive breast cancer from 1991 to 2014. Follow-up began at breast cancer diagnosis and ended at breast cancer recurrence (BCR), death, emigration, or June 8, 2020. The World Health Organization guidelines were used to classify BMI, waist circumference, and body fat percentage into three categories of healthy weight, overweight, and obesity. We fit Cox regression models to compute adjusted hazard ratios (HRs) with 95% confidence intervals (CI) of BCR according to body composition. To evaluate effect measure modification, we stratified Cox models by patient, tumor, and treatment characteristics.

**Results:**

In total, 263 BCRs were diagnosed over 12,816 person-years among 1099 breast cancer patients with a median follow-up of 11.1 years. Obesity according to BMI (HR = 1.44 [95%CI 1.00–2.07]), waist circumference (HR = 1.31 [95%CI 0.98–1.77]), and body fat percentage (HR = 1.41 [95%CI 1.02–1.98]) was associated with increased risk of BCR compared with healthy weight. Obesity was stronger associated with BCR in patients with low socioeconomic position (HR = 2.55 [95%CI 1.08–6.02]), larger tumors > 20 mm (HR = 2.68 [95%CI 1.42–5.06]), estrogen-receptor-negative breast cancer (HR = 3.13 [95%CI 1.09–8.97]), and with adjuvant chemotherapy treatment (HR = 2.06 [95%CI 1.08–4.31]).

**Conclusion:**

Higher pre-diagnostic BMI, waist circumference, and body fat percentage was associated with increased risk of BCR. The association between obesity and BCR appears dependent on patient, tumor, and treatment characteristics.

**Supplementary Information:**

The online version contains supplementary material available at 10.1186/s40170-023-00308-0.

## Introduction

Since the 1980s, advances in breast cancer detection and treatment have resulted in an increasing number of patients surviving breast cancer [1, 2]. Concurrently there has been an increase in obesity prevalence [3] with globally 110,000 breast cancer cases attributable to excess body weight in 2012 [4]. This has motivated researchers to investigate preventive public health interventions [5] and initiate trials with weight-loss interventions [6] to reduce the risk and improve the outcome of breast cancer. Yet, limited research has been carried out to identify characteristics of breast cancer patients that have negative health effects of their obesity. Obesity may be associated with poor breast cancer prognosis due to insufficient treatment [7]. For example, chemotherapy is dosed based on body surface area and the drug dosage determination guidelines include a praxis called dose-capping, meaning that a reduced, sub-optimal, chemotherapy dose may be prescribed to patients with obesity to minimize toxicities [7, 8]. Patients with greater body size can also be affected by underdosing due to underestimated body surface area [9]. Further, treatment with aromatase inhibitors may be less effective in breast cancer patients with obesity due to their inherently higher levels of aromatase [10, 11]. Obesity may also be associated with poor breast cancer prognosis due to metabolic changes. In individuals with obesity, white adipocytes are likely to become hypertrophic and hyperplastic, resulting in physiologic changes such as elevated FFAs and triglycerides, increased blood glucose, and insulin resistance. Further, obesity-associated adipose tissue produces more pro-inflammatory cytokines, such as tumor necrosis factor alpha, interleukin-6, interleukin-1, and adipokines. Through these mechanisms the presence of excess adipocytes and the obesity-induced change in adipose tissue may promote the early stages of metastasis [12–14]. To identify breast cancer patients with obesity at higher risk of disease recurrence and death, it is important to investigate whether patient, tumor and treatment characteristics can predict patient sub-populations in need of improved surveillance and/or treatment.

Body mass index (BMI) is still the standard tool for measuring and defining obesity [15]. Yet, BMI is controversial and its specificity questioned. Several other anthropometric measures have been suggested as more precise in determining obesity [15] and may be relevant to use systematically when assessing breast cancer patients for treatment.

The purpose of this study was to examine the association between pre-diagnostic anthropometric measures and breast cancer outcomes in the Malmö Diet and Cancer Study (MDCS). Further, the study aimed to assess patient, tumor and treatment characteristics associated with prognosis in breast cancer patients with obesity.

## Patients and methods

We conducted a prospective cohort study nested in the MDCS cohort.

### Data sources

The MDCS is a prospective cohort study that enrolled 17,035 women in Malmö, Sweden from 1991 to 1996. The primary objective of the MDCS was to investigate associations between dietary patterns and cancer risk. The MDCS enrolled 42.6% of the eligible population born between 1923 and 1950 [16]. The MDCS is updated annually with information on incident cancer cases and vital status through record linkage to the Swedish Cancer Registry, the Southern Swedish Regional Tumor Registry, and the Swedish Cause of Death Registry. All Swedish residents have a unique civil registration number in the National Population Register, allowing for 100% accurate data linking.

### Study population

**Fig. 1:**
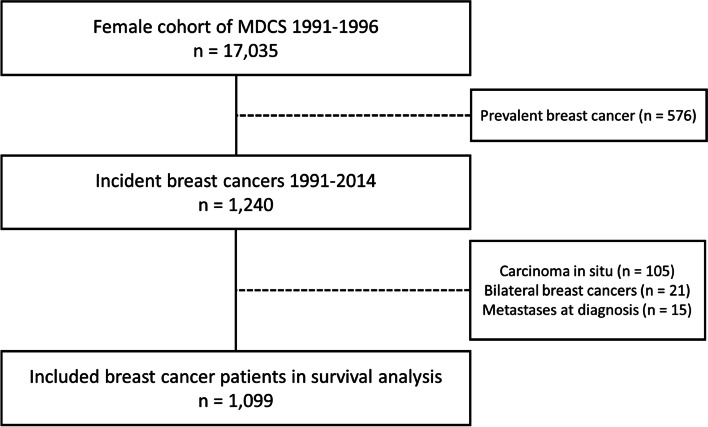
Flowchart of the study population. *MDCS = Malmö Diet and Cancer Study

Between 1991 and 2014, 1240 female MDCS participants were diagnosed with invasive breast cancer (Fig. 1). Participants with a breast cancer diagnosis before enrollment in MDCS were excluded as prevalent cases. We also excluded participants with carcinoma in situ*,* bilateral breast cancer or de novo metastatic disease. The final study population in the survival analyses included 1099 female breast cancer patients.

### Anthropometric measures

Trained research nurses collected anthropometric measures including, height, weight, waist circumference, and body fat percentage upon enrollment in the MDCS [16, 17]. Hence, the anthropometric data used in this study was collected at study baseline prior to the diagnosis of breast cancer. We used the World Health Organization’s definition of BMI in kg/m^2^, waist circumference in centimeters and body fat percentage to group the patients according to body composition [18]. Patients with a BMI of 18.5–24.9 kg/m^2^, a waist circumference below 81 cm, or a body fat percentage of 24% or lower were considered to have healthy weight. Patients with a BMI of 25.0–29.9 kg/m^2^, a waist circumference between 81 and 85 cm, or a body fat percentage exceeding 24% but below 32%, were considered to have overweight. Patients with a BMI of more than 30.0 kg/m^2^, a waist circumference of more than 85 cm, or a body fat percentage of 32% or more were considered to have obesity [18]. In the multivariable models, these variables were included as categorical variables with healthy weight as the reference group.

A body shape index (ABSI) has been suggested to predict mortality risk more precisely than alternative anthropometric measures. Therefore, we calculated ABSI according to the suggested formula by Krakauer and Krakauer [19]:

$$\frac{\mathrm{Waist\,Circumference}}{{\mathrm{BMI}}^{2/3}*{\mathrm{height}}^{1/2}}$$

In multivariable analyses, ABSI was divided into cohort-specific tertiles and the first tertile was used as the reference group.

BMI was used to determine body composition for descriptive purposes of patient characteristics in Table 1 and sensitivity analyses.

**Table 1:** Patient, tumor, and treatment characteristics according to body mass index in 1099 patients with incident invasive breast cancer in the Malmö Diet and Cancer Study

	Total	Healthy weight	Overweight	Obesity
	*N* = 1099	*N* = 556	*N* = 384	*N* = 159
**Age at diagnosis, median (IQR)**	66.3 (61.2–72.8)	64.9 (59.9–70.5)	68.8 (62.8–73.9)	69.2 (63.3–76.2)
**Menopausal status at diagnosis**				
Premenopausal	65 (6.4%)	40 (7.7%)	19 (5.5%)	6 (4.0%)
Postmenopausal	951 (93.6%)	478 (92.3%)	329 (94.5%)	144 (96.0%)
**Histological grade according to Nottingham score**				
Grade I, 3–5 p	269 (26.6%)	160 (31.2%)	76 (21.7%)	33 (22.4%)
Grade II, 6–7 p	478 (47.3%)	239 (46.6%)	162 (46.3%)	77 (52.4%)
Grade III, 8–9 p	263 (26.0%)	114 (22.2%)	112 (32.0%)	37 (25.2%)
**Tumor size, mm**				
< 10	178 (17.0%)	102 (19.0%)	57 (15.8%)	19 (12.7%)
10–20	568 (54.3%)	296 (55.1%)	196 (54.4%)	76 (50.7%)
> 20	301 (28.7%)	139 (25.9%)	107 (29.7%)	55 (36.7%)
**Nodal status**				
Negative	675 (68.3%)	366 (71.6%)	218 (64.9%)	91 (64.1%)
Positive	314 (31.7%)	145 (28.4%)	118 (35.1%)	51 (35.9%)
**Histological type**				
Invasive ductal cancer	714 (69.8%)	353 (68.1%)	256 (71.3%)	105 (71.9%)
Invasive lobular cancer	201 (19.6%)	110 (21.2%)	63 (17.5%)	28 (19.2%)
Other	108 (10.6%)	55 (10.6%)	40 (11.1%)	13 (8.9%)
**Estrogen receptor status**				
Negative	97 (10.1%)	48 (9.9%)	36 (10.8%)	13 (9.3%)
Positive	860 (89.9%)	436 (90.1%)	297 (89.2%)	127 (90.7%)
**Surgical procedure**				
Mastectomy	427 (42.1%)	225 (43.1%)	138 (39.9%)	64 (43.8%)
Breast conserving surgery	587 (57.9%)	297 (56.9%)	208 (60.1%)	82 (56.2%)
**Endocrine therapy, planned adjuvant**				
No	453 (42.7%)	247 (45.8%)	142 (38.4%)	64 (42.1%)
Yes	608 (57.3%)	292 (54.2%)	228 (61.6%)	88 (57.9%)
**Chemotherapy, planned adjuvant**				
No	844 (84.0%)	412 (82.7%)	305 (84.3%)	127 (87.6%)
Yes	161 (16.0%)	86 (17.3%)	57 (15.7%)	18 (12.4%)
**Radiotherapy, planned adjuvant**				
No	397 (39.4%)	199 (40.0%)	139 (38.2%)	59 (40.7%)
Yes	610 (60.6%)	299 (60.0%)	225 (61.8%)	86 (59.3%)
**Socioeconomic position**				
Low	302 (28.3%)	101 (18.8%)	132 (35.2%)	69 (44.8%)
Low middle	64 (6.0%)	28 (5.2%)	19 (5.1%)	17 (11.0%)
High middle	346 (32.4%)	193 (35.9%)	119 (31.7%)	34 (22.1%)
﻿ High	355 (33.3%)	216 (40.1%)	105 (28.0%)	34 (22.1%)
**Do you smoke?**				
Yes, I smoke regularly	270 (24.6%)	165 (29.7%)	76 (19.8%)	29 (18.2%)
Yes, I smoke occasionally	35 (3.2%)	21 (3.8%)	10 (2.6%)	4 (2.5%)
No, I have stopped smoking	333 (30.3%)	161 (29.0%)	122 (31.8%)	50 (31.4%)
No, I have never smoked	461 (41.9%)	209 (37.6%)	176 (45.8%)	76 (47.8%)

^a^*IQR* Interquartile range

### Socioeconomic position

Consistent with the Swedish socioeconomic classification [20], we categorized socioeconomic position (SEP) for descriptive purposes as *low* if patients had unskilled manual labor with < 2 years of post-high school education, *low-middle* if skilled manual labor with > 2 years of post-high school education, *high-middle* if assistant non-manual labor with < 3 years of post-high school education, and *high* if non-manual labor with ≥ 3 years of post-high school education. In multivariable models, SEP was dichotomized into low SEP vs high SEP. The low SEP group included patients categorized as low or low-middle and the high SEP group included patients categorized as high-middle and high.

### Definitions of analytic variables

Age was described as the median age at breast cancer diagnosis. In the multivariable models, age was included as a continuous variable defined at the time of breast cancer diagnosis.

We defined three categories of tumor size in millimeters (mm): less than 10 mm, 10–20 mm, and more than 20 mm. Tumor size was modeled as a categorical variable [21]. In sensitivity analyses, tumor size was modeled as a dichotomous variable (≤ 20 mm/ > 20 mm). The histological grade of breast cancer was assessed and described according to the Nottingham Histological Grade [22] and categorized as grade I, grade II, and grade III; this was modeled as a categorical variable. Menopausal status at diagnosis (premenopausal/postmenopausal), estrogen-receptor (ER) status (positive/negative), lymph node status (positive [present metastatic lymph nodes]/negative [no metastatic lymph nodes]), surgery (breast cancer conserving surgery/mastectomy), along with the intended adjuvant treatment with radiation (yes/no), chemotherapy (yes/no), and endocrine therapy (yes/no) were all treated as dichotomous variables.

### Outcome

The primary endpoint was breast cancer recurrence (BCR), defined as the time from breast cancer diagnosis until the earliest occurrence of invasive loco-regional recurrence or distant metastases. Trained medical doctors retrieved data on recurrence from medical charts of all patients diagnosed with breast cancer in the MDCS. The secondary endpoint was mortality, defined as the time from breast cancer diagnosis until death from any cause. Data on mortality was retrieved from the Swedish Cause of Death Registry.

### Follow-up and statistical analysis

Follow-up for BCR and mortality began at breast cancer diagnosis and continued until the first event of BCR, death, emigration or end of follow-up on June 8, 2020. Patients with these events were censored.

We used Cox regression models to compute crude and adjusted hazard ratios (HRs) with 95% confidence intervals (95%CI) for BCR and mortality. Only patients with complete data on all regressed variables were included in the analyses. The adjusted model included the following co-variables: age at diagnosis (continuous), node status (dichotomous), ER status (dichotomous), histological grade (categorical), tumor size (categorical), type of surgery (dichotomous), adjuvant radiotherapy (dichotomous), adjuvant chemotherapy (dichotomous), and adjuvant endocrine therapy (dichotomous). Complete case data were available for 941 patients, which were included in the adjusted models.

We performed a series of pre-planned sensitivity analyses using Cox regression models adjusted for the previously stated covariates excluding the stratification variable of interest to investigate the association between obesity and breast cancer prognosis. The reference group used for the stratified analyses was within strata, i.e., we compared healthy weight versus obesity among patients with low SEP. First, we examined differences in patient characteristics among healthy weight and breast cancer patients with obesity according to BMI in terms of BCR. Second, we investigated the prognostic value of tumor characteristics according to BMI in relation to BCR. Third, to examine any possible differences in treatment patterns according to BMI, we conducted analyses of BCR and BMI stratified by treatment with adjuvant chemotherapy, radiotherapy, endocrine therapy, and type of primary surgery. Finally, to assess whether the exposure of obesity combined with certain patient, tumor and treatment characteristics was greater than the individual effects alone, we conducted pre-planned interaction tests between obesity and the stratified factors that displayed a negative effect on the outcome. We used logistic regression models adjusted for the previously stated co-variates, dichotomous exposure to obesity, and the variable of interest, we estimated the relative excess risk due to interaction [23].

## Results

Among 1099 eligible patients, 263 BCRs were diagnosed over 12,816 person-years with a median follow-up of 11.1 years (interquartile range [IQR] was 6.6–16.2). The median time from collection of anthropometric measures until breast cancer diagnosis was 10.8 years (IQR 6.0–15.4). The cohort consisted of 556 patients with healthy weight, 384 patients with overweight and 159 patients with obesity. In the cohort, median BMI was 24.9 kg/m^2^ (IQR 22.8–27.7), median waist circumference was 77 cm (IQR 71–84), and the median body fat percentage was 31% (IQR 28–34). The median age at breast cancer diagnosis was 66.3 years (IQR 61.2–72.8). Patients with obesity were generally older than patients with healthy weight (Table 1).

Patients with overweight or obesity according to BMI were more often diagnosed with a higher histological grade, had larger tumors and positive nodal status at diagnosis compared with patients with healthy weight (Table 1). Invasive lobular breast cancer was more common among patients with healthy weight. Endocrine therapy, radiotherapy, and mastectomy were less frequently assigned to patients with overweight than to patients with healthy weight or obesity. However, chemotherapy was more frequently assigned to patients with overweight or obesity than to patients with healthy weight. Patients with obesity had lower SEP than patients with healthy weight, while patients with healthy weight were more likely to smoke than patients with overweight or obesity.

In multivariable analyses, having obesity according to BMI was associated with an increased rate of BCR (HR_adj_ = 1.56 [95%CI 1.00–2.46) and mortality (HR_adj_ = 1.51 [95%CI 1.05–2.18) in comparison to having healthy weight (Table 2). Having obesity according to waist circumference was also associated with a higher risk of BCR (HR_adj_ = 1.46 [95%CI 1.00–2.13]) and mortality (HR_adj_ = 1.45 [95%CI 1.07–1.96]). Patients with obesity according to body fat percentage were at higher risk of BCR (HR_adj_ = 1.41 [95%CI 1.02–1.98]), whereas the precision of the mortality estimate was lower (HR_adj_ = 1.30 [95%CI 0.74–2.27]). Having a high ABSI was not associated with increased risk of BCR (Tertile 3, HR_adj_ = 0.97 [95%CI 0.68–1.39]), but increased risk of mortality (Tertile 3, HR_adj_ = 1.24 [95%CI 0.91–1.71]) (Supplemental Table S1).

**Table 2:** Survival estimates of recurrence and mortality according to body mass index, waist circumference, and body fat percentage in 1099 patients with incident invasive breast cancer in the Malmö Diet and Cancer Study

Endpoint	Body composition	No. of patients	Events	Unadjusted HR (95%CI)	Adjusted HR^a^ (95%CI)
**Body mass index**					
**Recurrence**	Healthy weight	556	129	(ref)	(ref)
	Overweight	384	92	1.17 (0.89–1.54)	1.23 (0.86–1.76)
	Obesity	159	42	1.39 (0.98–1.99)	1.56 (1.00–2.46)
**Mortality**	Healthy weight	556	166	(ref)	(ref)
	Overweight	384	160	1.57 (1.26–1.96)	1.18 (0.88–1.59)
	Obesity	159	72	1.73 (1.31–2.29)	1.51 (1.05–2.18)
**Waist circumference**					
**Recurrence**	Healthy weight	723	166	(ref)	(ref)
	Overweight	119	33	1.34 (0.92–1.95)	1.18 (0.73–1.90)
	Obesity	223	59	1.35 (1.01–1.83)	1.46 (1.00–2.13)
**Mortality**	Healthy weight	723	229	(ref)	(ref)
	Overweight	119	45	1.26 (0.92–1.74)	1.03 (0.67–1.58)
	Obesity	223	113	1.71 (1.36–2.15)	1.45 (1.07–1.96)
**Body fat percentage**					
**Recurrence**	Healthy weight	109	31	(ref)	(ref)
	Overweight	476	101	0.96 (0.58–1.59)	0.99 (0.53–1.85)
	Obesity	514	131	1.25 (0.76–2.04)	1.41 (1.02–1.98)
**Mortality**	Healthy weight	109	31	(ref)	(ref)
	Overweight	476	151	1.39 (0.86–2.24)	1.25 (0.71–2.20)
	Obesity	514	216	1.97 (1.23–3.14)	1.30 (0.74–2.27)

^a^Age, histological grade, tumor size, nodal status, estrogen-receptor status, surgery, planned adjuvant radio-, chemo-, and endocrine therapy

^b^*HR* Hazard ratio, *CI* Confidence interval

**Fig. 2:**
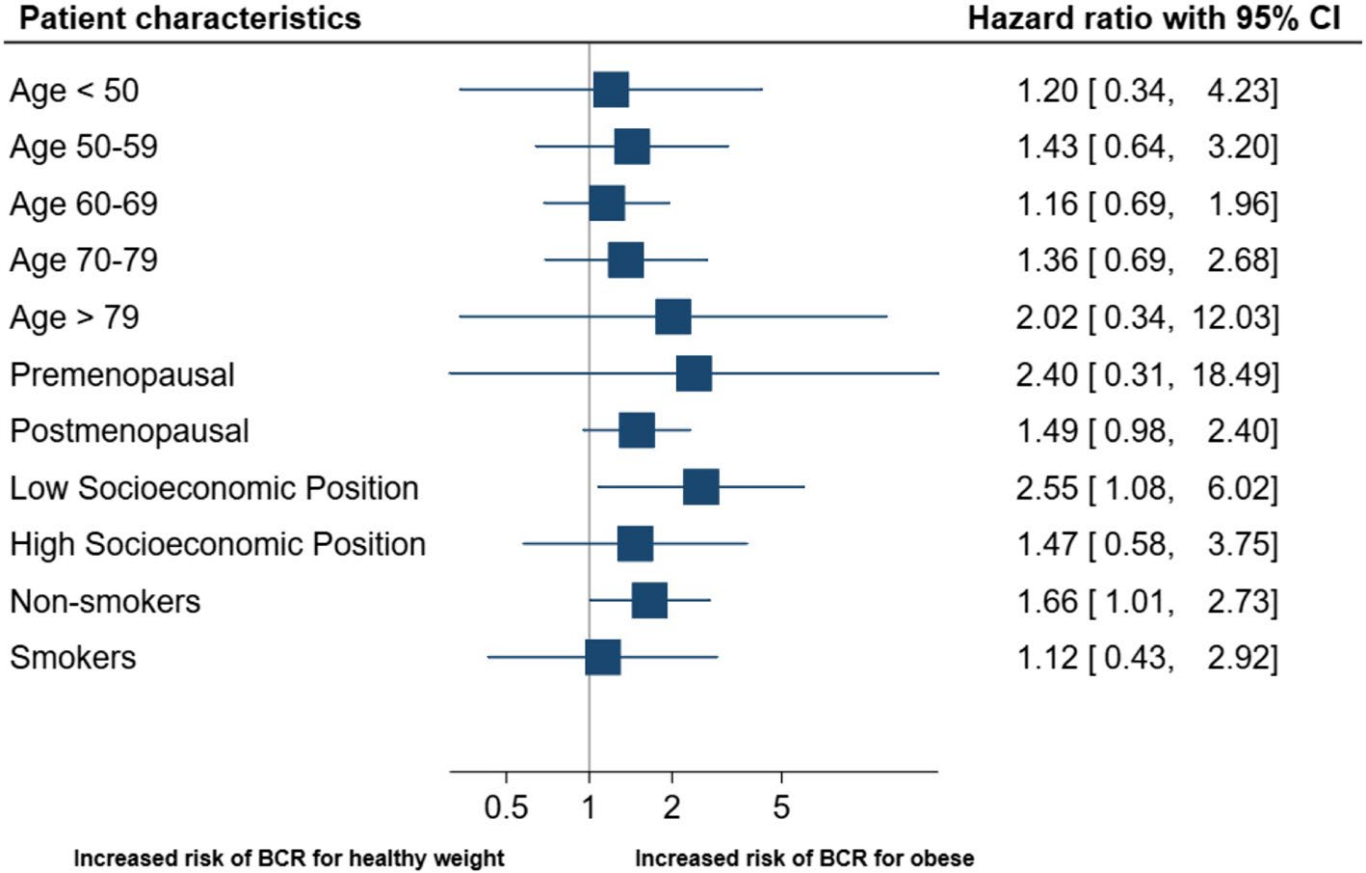
The relationship between patient characteristics and recurrence rates in breast cancer patients with healthy weight vs obesity defined by BMI. Forest plot shows adjusted HR (95% CI) of breast cancer recurrence for patients with obesity (BMI ≥ 30.0) compared with patients with healthy weight (BMI 18.5–24.9), according to stratified patient characteristics. *BCR = breast cancer recurrence. **The stratified analyses were adjusted for the following co-variables excluding the stratified variable of interest: age, histological grade, tumor size, nodal status, estrogen-receptor status, surgery, planned adjuvant radio-, chemo-, and endocrine therapy. ***The relative excess risk due to interaction with 95% CI for socioeconomic position was 0.94 (0.11–1.76) indicating a super-additive interaction effect, suggesting that the response to multimodal exposure are greater than the sum of the independent responses to low socioeconomic position or obesity alone

In Fig. 2, the association between body composition (BMI) and BCR is depicted according to patient characteristics. Having obesity was associated with BCR in postmenopausal women (HR_adj_ = 1.49 [95%CI 0.98–2.40]). Patients with obesity had an increased risk of BCR if they had low SEP (HR_adj_ = 2.55 [95%CI 1.08–6.02]) or if they were non-smokers (HR_adj_ = 1.66 [95%CI 1.01–2.73]). No association between having obesity and BCR was observed among patients with high SEP (HR_adj_ = 1.47 [95%CI 0.58–3.75]) or smokers (HR_adj_ = 1.12 [95%CI 0.43–2.92]).

**Fig. 3:**
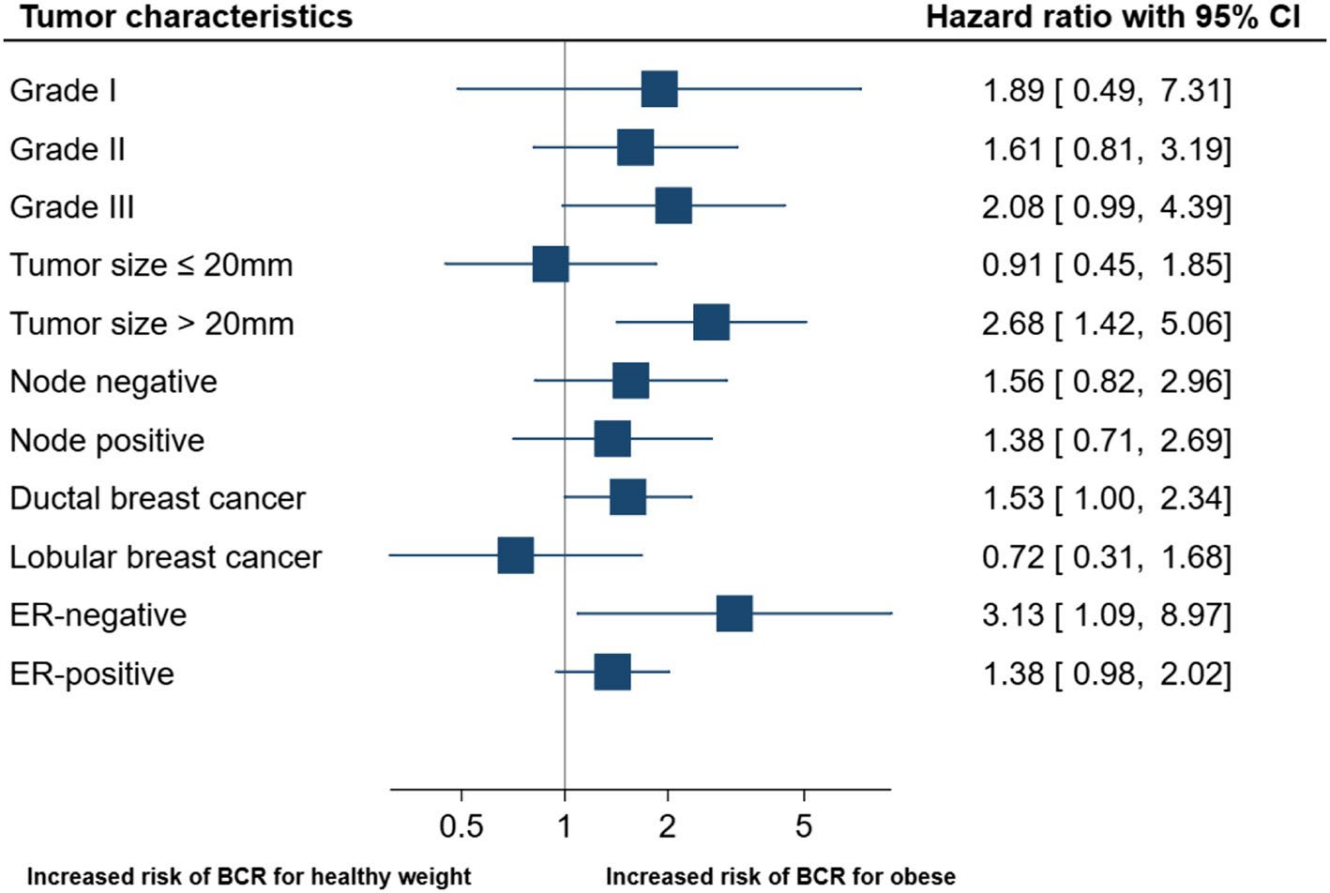
The relationship between tumor characteristics and recurrence rates in breast cancer patients with healthy weight vs with obesity defined by BMI. Forest plot shows adjusted HR (95% CI) of breast cancer recurrence for patients with obesity (BMI ≥ 30.0) compared with patients with healthy weight (BMI 18.5–24.9), according to stratified tumor characteristics. *BCR = breast cancer recurrence; ER = estrogen receptor. ** The stratified analyses were adjusted for the following co-variables excluding the stratified variable of interest: age, histological grade, tumor size, nodal status, estrogen-receptor status, surgery, planned adjuvant radio-, chemo-, and endocrine therapy. *** The relative excess risk due to interaction (RERI) with 95% CI for tumor size > 20 mm was 1.78 (− 0.16–3.72)”. The relative excess risk due to interaction with 95% CI for ER-negative disease was 0.79 (− 0.84–2.46). In this case, the RERI value is 1.78 and 0.79, which are greater than zero, indicating the presence of positive interaction between the risk factors (tumor size > 20 mm or ER-negative disease and obesity) on the outcome. However, the 95% CI for RERI includes zero. This suggests some uncertainty, and the possibility that there is actually no interaction between the two risk factors cannot be ruled out. A larger sample size may be needed

Figure 3 illustrates the relationship between body composition (BMI) and BCR based on tumor characteristics. Certain tumor characteristics were linked to the rate of recurrence, with histological grade III being associated with an increased risk of BCR in breast cancer patients with obesity (HR_adj_ = 2.08 [95%CI 0.99–4.39]). No association between obesity and BCR was observed for patients with histological grade I (HR_adj_ = 1.89 [95%CI 0.49–7.31]) or histological grade II (HR_adj_ = 1.61 [95%CI 0.81–3.19]). In comparison to patients with healthy weight, patients with obesity who had tumors larger than 20 mm at breast cancer diagnosis were at increased risk of BCR (HR_adj_ = 2.68 [95%CI 1.42–5.06]). However, patients with obesity and tumors of 20 mm or less were not at increased risk of BCR (HR_adj_ = 0.91 [95%CI 0.45–1.85]). Patients with obesity who were diagnosed with ductal breast cancer had increased risk of BCR (HR_adj_ = 1.53 [95%CI 1.00–2.34]), while no association was observed among patients diagnosed with lobular breast cancer (HR_adj_ = 0.72 [95%CI 0.31–1.68]). Patients with obesity and ER-negative disease had an increased risk of BCR (HR_adj_ = 3.13 [95%CI 1.09–8.97]) compared with patients with healthy weight. Similarly, patients with obesity and ER-positive disease had increased risk of BCR compared with patients with healthy weight (HR_adj_ = 1.38 [95%CI 0.98–2.02]).

**Fig. 4:**
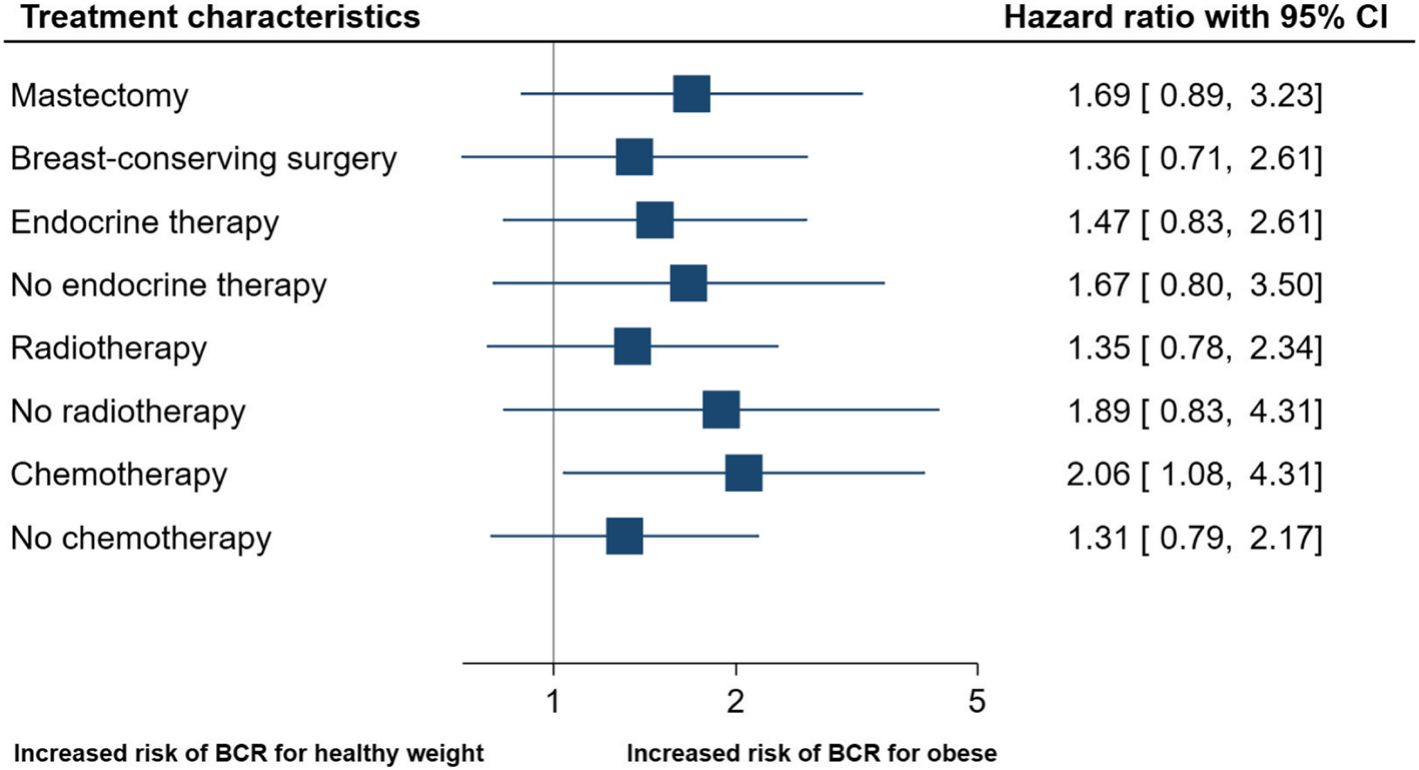
The relationship between treatment characteristics and recurrence rates in breast cancer patients with healthy weight vs obesity defined by BMI. Forest plot shows adjusted HR (95% CI) of breast cancer recurrence for patients with obesity (BMI ≥ 30.0) compared with patients with healthy weight (BMI 18.5–24.9), according to stratified treatment characteristics. *BCR = breast cancer recurrence. ** The stratified analyses were adjusted for the following co-variables excluding the stratified variable of interest: age, histological grade, tumor size, nodal status, estrogen-receptor status, surgery, planned adjuvant radio-, chemo-, and endocrine therapy. ***The relative excess risk due to interaction with 95% CI for chemotherapy in patients with obesity was 1.21 (0.95–3.37) indicating a super-additive interaction effect, suggesting that the response to these exposures combined is greater than the sum of the independent responses to chemotherapy and obesity alone

Breast cancer patients with obesity who were assigned to adjuvant chemotherapy had a higher risk of BCR compared with breast cancer patients with a healthy weight (HR_adj_ = 2.06 [95%CI 1.08–4.31]). Patients not assigned to chemotherapy were not at a higher risk of BCR (HR_adj_ = 1.31 [95%CI 0.79–2.17]) (Fig. 4).

## Discussion

This study showed an association between obesity and inferior breast cancer outcomes. We identified several characteristics that indicate a worse prognosis among patients with obesity. Low SEP was associated with a poor breast cancer prognosis among breast cancer patients with obesity compared with patients with healthy weight. Tumor characteristics, such as histological grade III, a tumor larger than 20 mm, ductal histology, or ER-negative tumors were associated with poorer prognosis in breast cancer patients with obesity compared with patients with healthy weight. Finally, patients with obesity who were assigned to treatment with chemotherapy had a poorer prognosis compared with patients with healthy weight who were assigned chemotherapy.

BMI has been criticized as a tool for determining obesity since it does not differentiate between fat tissue and other body tissues, such as muscle tissue [24, 25]. Given ethnic and geographical variation in the relationship between fat tissue and other body tissues globally [26], it has been suggested that anthropometric measures other than BMI should be used to better predict obesity and its impact on disease [15, 27, 28]. Waist circumference more specifically indicate central obesity compared with BMI [29] and central obesity has been suggested a better predictor of inferior breast cancer outcome than peripheral obesity [30]. Body fat percentage is another anthropometric measure that has been suggested to have disease prognostic value [31]. However, in comparison to BMI and waist circumference, which can be collected using a standard weight and measurement, evaluating body fat percentage is more complex and requires a bioelectrical impedance analyzer. Few studies have investigated the relationship between body fat percentage and breast cancer outcomes [32]. ABSI has recently been suggested to better predict obesity-related mortality than BMI [33], but few studies have investigated its relationship to breast cancer prognosis. Our findings confirm previously reported mortality predictions [33]. Nevertheless, when adjusting for breast cancer prognostic factors or predicting BCR, ABSI added no value. In this study, all anthropometric measures served as prognostic tools for breast cancer outcome; as the measures were comparably associated with breast cancer outcome. However, no comparative statistical tests were used to assess the prognostic difference of the anthropometric measures.

Previous investigations studying the impact of low SEP on breast cancer outcome suggested that low SEP was associated with higher mortality [34], but not BCR indicating potential under-detection of BCR among patients with low SEP [35]. In this study, the association between obesity and poor prognosis was predominantly present among breast cancer patients with low SEP regardless of whether studying recurrence or mortality, while no association between obesity and poor prognosis was evident among patients with high SEP. Reasons underlying these findings are not clear, but may be partly explained by differences in access to healthcare and adherence to treatment and follow-up [36]. Low SEP is associated with a poorer diet, physical inactivity [37, 38], and higher comorbidity, which may contribute to a worse prognosis [39]. Yet, other explanations as to why obesity might be a mediator for social inequality in breast cancer outcomes may exist.

The observation that non-smoking patients with obesity have a poorer prognosis than smoking patients with obesity is likely to be due to misclassification and residual confounding in the association and indicates presence of the obesity paradox when stratifying analyses for smoking status [40].

The observed association between having obesity and increased risk of BCR in breast cancer patients with ER-positive disease agrees with previous research [41] and was recently highlighted by the *International Association for Research on Cancer* [42]. While previous research has established that obesity is associated with poorer outcomes in ER-positive breast cancer, our study suggests that this association may be even stronger in estrogen-independent breast cancer, and add novel insights to the relationship between breast cancer subtypes, obesity, and clinical outcomes. Recent research supports our findings and suggests that obesity is also a prognostic factor in hormone-independent breast cancer [43, 44].

The observed increased risk of BCR and mortality among women with obesity assigned chemotherapy treatment reflects previous studies [45, 46]. Despite existing literature supporting the safety of weight-based doses of chemotherapy regardless of weight-status [8] dose-capping in patients with obesity can occur [47, 48]. No data on chemotherapy dosing was available in this study. Therefore, we were unable to determine if patients with obesity received the optimal dose of chemotherapy. Dose-capping might thereby have contributed to our findings [7]. It is important to highlight this potentially suboptimal treatment with chemotherapy and to call on future studies to ensure optimal healthcare regardless of body composition [49].

We observed no difference in the risk of BCR in patients with obesity who were treated with endocrine therapy versus those who were not. We were unable to stratify by the type of endocrine therapy. As a result, those treated with tamoxifen may conceal any possible association between poor prognosis and obesity in breast cancer patients treated with aromatase inhibitors.

Our study has limitations. The patients’ anthropometric measures were obtained upon cohort-enrollment, not at breast cancer diagnosis. As anthropometric measures like BMI can fluctuate over time, our study results may be prone to misclassification bias [50]. Conclusions on intervention strategies for breast cancer survivors cannot be developed based on findings of this study as data on anthropometric measures were collected prior to breast cancer diagnosis. Intervention strategies to improve breast cancer prognosis are generally assigned to the time of diagnosis or during the post-diagnostic adjuvant treatment plan [51]. Similarly, information on SEP was retrieved upon enrollment in the MDCS, and may be misclassified. However, this is unlikely as participants in the MDCS were at least 40 years old at inclusion, and the yearly rate of workplace change for Swedish women over 40 is 5% [52]. Some studies have linked dietary [53] and physical activity [54] habits to the prognosis of breast cancer. We do not account for these possible confounders in our models, which should be considered when interpreting the results of this study. Further, our study is limited by small sample size and the findings; therefore, need to be validated in larger cohorts. Finally, the adjustment for co-variates in multivariable models attenuated the associations between some anthropometric measures and outcomes; therefore, our study might be prone to residual confounding.

With an ongoing obesity epidemic worldwide, having overweight or obesity will become more common than having healthy weight [55], it is therefore pertinent to identify populations of breast cancer patients with overweight or obesity who have a poor prognosis. Such information could be used to optimize treatment for all breast cancer patients, regardless of body composition. This study has highlighted several patient populations with obesity who may have a higher treatment demand than others when diagnosed with breast cancer. Yet, the associations observed in this study needs to be confirmed in larger cohorts with more up-to-date data.

## Conclusion

Obesity defined by high pre-diagnostic levels of BMI, waist circumference and body fat percentage were associated with an increased risk of recurrence and mortality among breast cancer patients. In this study, breast cancer patients with obesity and low SEP, a large breast tumor, breast cancer of high histological grade, ER-negative breast cancer, and/or patients intended to be treated with chemotherapy were at higher risk of BCR and mortality compared to similar patients with healthy weight. The identification of these vulnerable patient groups among breast cancer patients with obesity may help guide researchers to patient populations in need of improved screening and/or treatment interventions.

## Supplementary Information

**Additional file 1:** **Supplementary Table S1.** A body shape index in relation to breast cancer recurrence and mortality.

## Data Availability

The data that support the findings of this study are available to appropriate academic parties upon reasonable request to the *Malmö Preventative Project/Malmö Diet and Cancer Study/ Malmö Offspring Study* steering committee (please see https://www.malmo-kohorter.lu.se/malmo-cohorts).

## Abbreviations

BCR: Breast cancer recurrence
BMI: Body mass index
ABSI: A Body Shape Index
SEP: Socioeconomic position
MDCS: Malmö Diet and Cancer Study
ER: Estrogen receptor
HR: Hazard ratio
CI: Confidence interval
IQR: Interquartile range
