# Supplementary material for: Obesity and breast cancer prognosis: pre-diagnostic anthropometric measures in relation to patient, tumor, and treatment characteristics
Source: Cancer Metab. 2023 Jun 27;11:8. doi: 10.1186/s40170-023-00308-0 (PMC10294507; doi:10.1186/s40170-023-00308-0)
Supplement: Supplementary file 1 — Additional file 1: Supplementary Table S1. A body shape index in relation to breast cancer recurrence and mortality. [file 40170_2023_308_MOESM1_ESM.docx]

**Supplementary Tables**

**Supplementary Table 1–** A body shape index in relation to breast cancer recurrence and mortality.

| **Endpoint** | **Tertiles of ABSI** | **No. of patients** | **Events** | **Unadjusted HR (95%CI)** | **Adjusted HR* (95%CI)** |
| --- | --- | --- | --- | --- | --- |
| **Recurrence** |  |  |  |  |  |
|  | Tertile 1 | 366 | 97 | (ref) | (ref) |
|  | Tertile 2 | 366 | 85 | 0.90 (0.67-1.21) | 0.98 (0.69-1.39) |
|  | Tertile 3 | 366 | 80 | 0.85 (0.63-1.14) | 0.97 (0.68-1.39) |
| **Mortality** |  |  |  |  |  |
|  | Tertile 1 | 366 | 114 | (ref) | (ref) |
|  | Tertile 2 | 366 | 132 | 1.22 (0.94-1.57) | 1.17 (0.85-1.61) |
|  | Tertile 3 | 366 | 151 | 1.38 (1.08-1.78) | 1.24 (0.91-1.71) |

*****Age, histological grade, tumor size, nodal status, estrogen-receptor status, surgery, adjuvant radiotherapy, chemotherapy, and endocrine therapy
